# ImVoxelGNet: Image to voxels geometry-aware projection for multi-view RGB-based 3D object detection

**DOI:** 10.1371/journal.pone.0320589

**Published:** 2025-05-19

**Authors:** Gang Xu, Biao Leng, Zhang Xiong

**Affiliations:** School of Computer Science and Engineering, Beihang University, Beijing, China; Bayer Crop Science United States: Bayer CropScience LP, UNITED STATES OF AMERICA

## Abstract

3D object detection based solely on image data presents a significant challenge in computer vision, primarily due to the need to integrate geometric perception processes derived from visual inputs. The key to overcoming this challenge lies in effectively capturing the geometric relationships across multiple viewpoints, thereby establishing strong geometric priors. Current methods commonly back-project voxels onto images to align voxel-pixel features, yet during this process, pixel features are insufficiently involved in learning, leading to a decrease in geometric perception accuracy and, consequently, impacting detection performance. To address this limitation, we propose a novel network framework called ImVoxelGNet. This framework first integrates features projected onto pixels via a expansion operation, compensating for the pixel information inadequately utilized in traditional back-projection methods, thus enabling more precise learning of spatial geometric features. Additionally, we design an implicit geometric perception structure that further refines the spatial geometric features obtained after integrating image features, learning the occupancy relationships in spatial voxels and updating them within the spatial features. Finally, we generate the final prediction results by combining a detection head with 3D convolutions. Evaluation on the ScanNetV2 multi-view 3D object detection dataset demonstrates that ImVoxelGNet achieves a performance improvement of up to 2.2% in mean average precision (mAP). This improvement effectively demonstrates the efficacy of our method in significantly enhancing 3D object detection performance through improved geometric perception and comprehensive scene understanding. Codes and data are released in https://github.com/xug-coder/ImVoxelGNet.

## Introduction

3D object detection plays a pivotal role in indoor scene understanding and serves as the foundation for a wide range of applications, including robotics, augmented reality, and autonomous systems [1–3]. Accurate 3D detection enables machines to better perceive and interact with their environment, which is critical for tasks such as navigation, manipulation, and scene reconstruction. For instance, in robotics [2], precise 3D detection facilitates safe and efficient movement within dynamic environments. Given that indoor environments [4] are typically dense with objects and structurally diverse, a robust 3D detection system has the potential to transform the way technology interfaces with real-world scenarios. Although recent advances, particularly those integrating deep learning and multi-view approaches, have demonstrated promise in this field, achieving reliable and efficient 3D detection remains an ongoing challenge. Addressing these issues requires innovative approaches that can effectively fuse multi-view data and improve 3D object detection performance in indoor environments [5,6].

In indoor environments [7–12], multi-view 3D object detection has emerged as a promising approach for accurately inferring the 3D structure of objects by leveraging multiple 2D images captured from different viewpoints. A common strategy [13,14] involves projecting the 2D features from these images into a shared 3D space, thereby enabling the reconstruction of object shape, position, and orientation. This process typically includes stages such as feature extraction, multi-view aggregation, 3D feature processing, and 3D bounding box prediction. By integrating information from multiple views, multi-view 3D detection has the potential to address challenges faced by single-view methods, such as occlusion, scale variation, and depth perception ambiguities [15,16]. In the multi-view feature aggregation process, existing methods widely utilize the back-projection operation. Specifically, a 3D voxel grid is defined in space, and the corresponding features are projected back onto 2D images using camera intrinsic and extrinsic parameters. After removing invalid correspondences, such as those outside the image boundaries, a mapping relationship is established between the 2D image features and the 3D voxel features. By aggregating features from multiple views into a shared voxel space, the 3D feature aggregation is completed. Following this, through a series of multi-layer convolutions and other operations on the 3D features, a 3D detection head is employed to generate the final output.

However, there are two potential risks and issues inherent in this process. First, the back-projection operation introduces certain challenges. This process involves mapping voxels onto the image plane, and this mapping is neither injective nor surjective. Unused voxels are determined by geometric constraints, which is reasonable, but not all voxels can be fully utilized. After projecting the voxels onto the image, the rounding operation results in a significant portion of the image pixels being underutilized, potentially leading to voxel-pixel mismatches or missed correspondences. Second, in the process of constructing the voxel grid and then directly applying 3D convolutions to learn the final detection results, there is a risk of insufficiently exploiting the latent geometric information and geometric features. Without fully incorporating this geometric context, the model’s 3D perception may become incomplete or inadequate, which could negatively impact the final detection performance. This lack of effective geometric feature utilization can lead to suboptimal 3D object detection results.

To address these two issues, we propose the ImVoxelGNet framework. Our approach is as follows:1)Enhanced Pixel-to-Voxel Mapping: We moderately expand the sampling range of pixel features mapped to voxel features. We hypothesize that the back-projection process, due to rounding operations and factors such as motion blur, refraction, and noise during image acquisition, introduces certain deviations. However, these deviations are generally constrained to a small region near the theoretical projection location. Based on this assumption, we sample and average pixel features in a neighborhood around the theoretical projection location to derive a new approximate value. This approximate value is then assigned as the feature value for the corresponding voxel position.2)Implicit Geometric Perception in Voxel Space: After constructing the voxel grid, we enhance geometric perception by learning spatial occupancy predictions for each voxel location through convolution operations on spatial features. These occupancy predictions are then multiplied element-wise with the voxel features, producing geometry-aware spatial features. This implicit geometric perception enables the model to better understand the spatial context and improve 3D feature representation.

Specifically, we designed a weighted averaging feature expansion method. After mapping spatial voxels onto the image plane, we select the pixel features in the vicinity of each projected position and compute a weighted average to determine the feature value for the corresponding spatial voxel. To evaluate the effectiveness of this approach, we experimented with various weighting strategies and neighborhood sizes as part of an ablation study. Ultimately, we adopted a learnable eight-neighborhood weighted kernel for optimal performance. In addition, we introduced an implicit geometric perception module after constructing the voxel grid. This module learns spatial features through 3D convolutions over the voxel grid and predicts the spatial occupancy at each voxel position. The predicted occupancy is then multiplied element-wise with the geometric features to produce geometry-aware spatial features. Finally, the geometry-aware voxel features are passed through a 3D object detection head that performs further convolutions to generate the final detection results. This pipeline ensures that both feature expansion and spatial geometric context are fully leveraged, resulting in improved 3D object detection performance.

Our contributions can be summarized as follows:

- We refine certain details in the back-projection process by fully leveraging different features obtained from projecting voxels back onto the images. This improvement contributed to enhanced performance.
- We design a geometry-aware module that enhances network performance by effectively utilizing geometric cues.
- We conduct extensive experiments to validate the effectiveness of our proposed strategies, demonstrating significant improvements over baseline methods and establishing the robustness of our approach.

## Method

**Fig 1:**
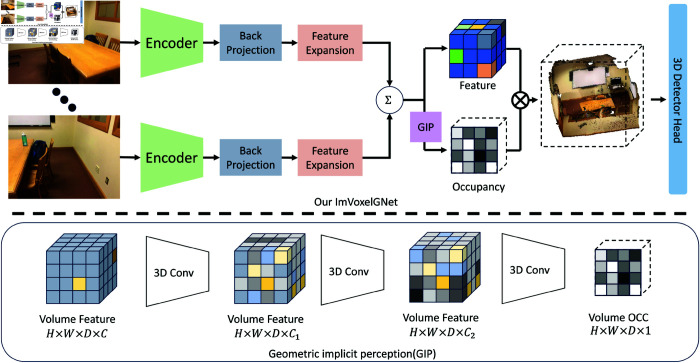
Pipeline of our, beginning by a 2D feature extractor from the input images, which are then expanded by feature expansion module and projected into a 3D voxel space. With GIP module, our enhance geometric perception.

In this section, we present a comprehensive introduction of the proposed ImVoxelGNet network model and methodology. The workflow of our network is depicted in Fig 1.

### Motivation

#### Inputs and goals.

Our method processes a collection of images $I_{n}\in {\mathbb{R}}^{W\times H\times 3}$ as input, where each image is characterized by its intrinsic and extrinsic parameters and can have an arbitrary resolution. Here, *t* denotes the *n*-th image in a set of *N* images. This work specifically targets multi-view scenarios, where *T*>1. The objective is to predict multiple 3D object bounding boxes using the proposed ImVoxelGNet network framework. Each bounding box is parameterized by (x,y,z,w,h,l), where (x,y,z) represents the spatial coordinates of the bounding box center, and *w*, *h*, and *l* define its width, height, and length, respectively.

#### Rgb-based multi-view 3D detection.

We provide an analysis of current image-based 3D object detection techniques in the context of scene understanding [4,13,17–19] and contrasted them with several well-known point cloud-based methods [20–22].

Point cloud-based methods naturally incorporate explicit 3D spatial information in their input data, enabling them to bypass the need for reconstructing 3D structures from images or other sources. This capability minimizes potential reconstruction errors and generally leads to more accurate and reliable outcomes. Some methods, while not using point cloud data directly as input, explicitly reconstruct geometric relationships across multiple views and perform detection based on the reconstructed point cloud. These approaches often leverage 3D point cloud data as a supervisory signal, aiding the network in learning geometric structures effectively.

In contrast, the method introduced in our research takes 2D RGB images as input and performs 3D object detection directly within the 2D image space. Although this design adds complexity to the network’s learning process, it eliminates the dependency on point cloud data, significantly reducing constraints on the input data. This design choice not only enhances the flexibility of the approach in terms of data requirements but also facilitates scalability to larger training datasets. Consequently, our method seeks to balance performance with accessibility, making it a viable solution for 3D object detection in scenarios where point cloud data is either unavailable or difficult to obtain.

While our approach introduces additional challenges in terms of learning and inference, it demonstrates strong potential for scalability with large datasets, offering a promising direction for further research and development in the field of 3D object detection.

### Back-projection

We adopt the back-projection steps outlined in [13,17]. Let $I_{t}\in {\mathbb{R}}^{W\times H\times 3}$ represent the *t*-th image in a sequence of *T* images. Our study specifically focuses on scenarios involving more than five images to detect 3D objects in each scene. Following the methodology of Murez *et al*. [13], we begin by extracting two-dimensional (2D) features from each input image using a pre-trained 2D backbone. This process produces four feature maps with resolutions of $\frac{W}{4}\times \frac{H}{4}\times c_{0}$, $\frac{W}{8}\times \frac{H}{8}\times 2c_{0}$, $\frac{W}{16}\times \frac{H}{16}\times 4c_{0}$, and $\frac{W}{32}\times \frac{H}{32}\times 8c_{0}$, respectively. These feature maps are aggregated using a Feature Pyramid Network (FPN), resulting in a unified tensor *F*_*t*_ with dimensions $\frac{W}{4}\times \frac{H}{4}\times c_{1}$. Here, *c*_0_ and *c*_1_ are backbone-specific parameters detailed in the implementation section.

The resulting 2D features *F*_*t*_ are then back-projected into a 3D voxel space $V_{t}\in {\mathbb{R}}^{N_{x}\times N_{y}\times N_{z}\times c_{1}}$. The orientation of this 3D voxel volume aligns the *z*-axis perpendicular to the ground, the *x*-axis pointing forward, and the *y*-axis orthogonal to both *x* and *z*. Spatial boundaries $x_{\min},x_{\max},y_{\min},y_{\max},z_{\min},z_{\max}$ are estimated empirically, following [13,21,23]. The relationship between the number of voxels and spatial extent is defined by the voxel size *s* as:


$$N_{x}s=x_{\max}-x_{\min},\;N_{y}s=y_{\max}-y_{\min},\;N_{z}s=z_{\max}-z_{\min}.$$

A pinhole camera model is used to establish correspondences between 2D coordinates (u,v) in the feature map *F*_*t*_ and 3D coordinates (x,y,z) in the voxel volume $V_{t}$. The relationship is defined as:


$$[\begin{array}{c} u\\ v \end{array}]=\Pi [\begin{array}{ccc} \frac{1}{4} & 0 & 0\\ 0 & \frac{1}{4} & 0\\ 0 & 0 & 1 \end{array}]KR_{t}[\begin{array}{c} x\\ y\\ z\\ 1 \end{array}],$$

where *K* and *R*_*t*_ represent the intrinsic and extrinsic camera matrices, respectively, and Π denotes the perspective projection. After projecting 2D features into 3D space, all voxels along the same camera ray inherit identical features.

Additionally, a binary mask *M*_*t*_, matching the dimensions of $V_{t}$, is created to indicate whether a voxel falls within the camera frustum. For a given image *I*_*t*_, the mask *M*_*t*_ is defined as:


$$M_{t}(x,y,z)=\left\{ \begin{array}{cc} 1, & \text{if }0\leq u<\frac{W}{4}\text{ and }0\leq v<\frac{H}{4},\\ 0, & \text{otherwise}. \end{array}\right.$$

Subsequently, the 2D feature map *F*_*t*_ is projected onto the 3D voxel grid $V_{t}$ for all valid voxels, according to:


$$V_{t}(x,y,z)=\left\{ \begin{array}{cc} F_{t}(u,v), & \text{if }M_{t}(x,y,z)=1\\ 0, & \text{otherwise}. \end{array}\right.$$

The final step involves aggregating the binary masks $M_{1},\ldots ,M_{t}$ to form a composite mask *M*, computed as:


$$M(x,y,z)=\left\{ \begin{array}{cc} \sum_{t}M_{t}(x,y,z), & \text{if }\sum_{t}M_{t}(x,y,z)>0\\ 1, & \text{otherwise}. \end{array}\right.$$

Ultimately, the 3D voxel volume V is synthesized by averaging the projected features across the valid voxels in the individual volumes $V_{1},\ldots ,V_{t}$, following the formula:


$$V=\frac{1}{M}\sum_{t}M_{t}V_{t}.$$

### Feature expansion

Undoubtedly, 3D detection approach based on multiple RGB images requires reconstructing and understanding the 3D space during the feature extraction and representation stages. This can be achieved through various implicit or explicit methods, enabling more accurate 3D detection results.

**Fig 2:**
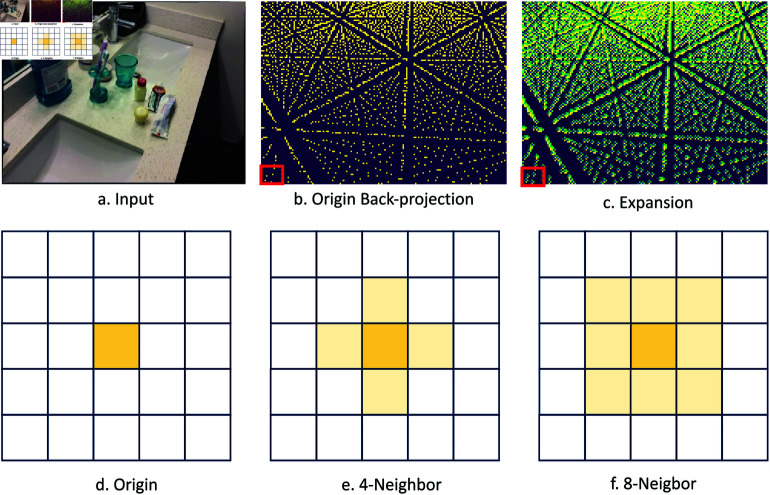
Feature expansion operation. We visualize the (a) input, (b) origin sampled pixels and (c) pixels with expansion. We also illustrate the (d) origin sampled pixel, (e) 4-neighbor and (f) 8-neighbor methods.

We conducted a thorough examination of the specific steps involved in the back-projection process. Back-projection is a commonly used operation for projecting image features into voxel space[13,17]. However, during this process, voxels are projected onto the image plane, which does not guarantee a surjective mapping from image pixels to voxels, or vice versa. As a result, many image regions do not contribute to the parameter updates in the latter stages of the network, as illustrated in Fig 2(a) and (b). To address this issue, we propose a method that incorporates additional image features into the projection process, as shown in (c), thereby improving the network’s 3D perception capabilities.

Specifically, we explored how to better utilize the points projected from voxels onto images by integrating convolutional operations. We began by proposing a hypothesis: during multi-view matching, factors such as scene dynamics, device vibrations, scene noise, and imaging noise may introduce certain errors in the correspondence between voxels and pixels derived from camera intrinsic and extrinsic parameters. In other words, the mapping from voxels to pixels may have slight inaccuracies. However, given the nature of these errors, their range is typically limited and usually confined to a small neighborhood around the projected pixel.

Building on this, we propose to incorporate the features of surrounding pixels in the vicinity of the projected pixel. To achieve this, the process can essentially be viewed as convolving the pixel with a convolutional kernel. We experimented with several approaches for this integration:

- Fixed convolutional kernel parameters:In this approach, we used a predefined convolutional kernel to aggregate the features from the projected pixel and its neighbors.
- Learnable convolutional kernels: Here, the convolutional kernel parameters were learned during training, allowing the model to adaptively determine the importance of surrounding pixels.

Initially, we tested using the four neighboring pixels (top, bottom, left, and right, as illustrated in (e) Fig 2) as well as the eight pixels(as illustrated in (f) Fig 2) in the immediate surrounding area for fixed convolutional kernel parameters. Finally, we experimented with a scientifically designed convolutional kernel tailored to balance computational efficiency and feature aggregation.

Through these methods, we aimed to enhance the accuracy of feature utilization in the back-projection process, ultimately improving the model’s overall performance in 3D perception.

### Geometric implicit perception

To enhance the system’s ability to comprehend spatial and geometric properties of the environment, we integrated an additional geometric implicit perception(GIP) module, as shown in Fig 1. This module is specifically designed to provide an implicit understanding of geometric occupancy within the voxel space, allowing the network to reason more effectively about spatial arrangements and the presence of geometric structures. The process begins with the spatially constructed geometric voxel grid, where the voxel features are flattened into a matrix representation of size H×W×D×C, with H,W,D denoting the shape of voxels and *C* = 256 for the length of features. This representation encapsulates the rich spatial and geometric information encoded within the voxel grid, serving as a compact but descriptive input to the subsequent processing steps.

Next, we designed three sequentially connected 3D convolutional modules. By applying three successive convolutions, we transformed the feature tensor of size HWDC into a prediction tensor of size HWD1 representing voxel classification. This prediction tensor can be directly multiplied with the original voxel features, thereby implicitly incorporating the spatial occupancy relationships. Importantly, the classification process leverages the high-dimensional feature representation of the voxels, allowing the network to capture subtle geometric cues that may not be immediately apparent in lower-dimensional feature spaces. This probabilistic assignment provides a nuanced understanding of voxel occupancy, enabling the network to identify and prioritize regions in the voxel space that are most likely to correspond to meaningful spatial entities.

The output probabilities from this module are subsequently integrated into the overall pipeline, where they play a crucial role in enhancing the network’s perception capabilities. By explicitly reasoning about the likelihood of voxel occupancy, the geometric perception module improves the system’s ability to model the spatial distribution of objects and surfaces. This enhancement is particularly valuable for tasks such as 3D object detection, where understanding spatial relationships and geometric configurations is critical for accurate localization and classification.

Moreover, the geometric perception module provides additional benefits by enriching the system’s feature representation. The probabilistic outputs serve as an intermediate supervisory signal, guiding the network to focus on geometrically relevant regions and filter out less significant areas of the voxel space. This leads to a more efficient allocation of computational resources and improved generalization to complex, unstructured 3D environments. Furthermore, the probabilistic approach adopted by this module introduces an element of uncertainty estimation, which can be leveraged to quantify the confidence of the network’s predictions. Such uncertainty measures are particularly useful in real-world applications, where noisy or incomplete data is common.

In summary, the geometric perception module significantly enhances the network’s spatial reasoning capabilities by providing an implicit yet robust understanding of voxel occupancy. Through the use of high-dimensional feature representation, multi-layer perceptrons, and probabilistic classification, this module contributes to a more accurate, efficient, and interpretable 3D perception system. Its integration into the overall pipeline not only improves the accuracy of 3D object detection but also establishes a foundation for further advancements in 3D scene understanding and reasoning.

## Experiments

### Experiment settings

#### Implement details.

Our ImVoxelGNet framework is implemented using the PyTorch deep learning library, alongside MMDetection3D [24], a modular and flexible toolbox for object detection. The implementation is conducted on a high-performance Linux workstation configured with an Intel E5-2640v4 CPU and four Nvidia GTX1080Ti GPUs, each equipped with 11GB of memory. This computational setup provides the necessary resources for handling the complexity and scale of the proposed framework, ensuring efficient training and inference.

Our neural network is trained on the dataset for 12 epochs, utilizing the AdamW optimizer, which is well-suited for modern deep learning applications due to its adaptive learning rate and weight decay capabilities. The training procedure closely adheres to the configuration detailed in [17], ensuring methodological consistency with established baselines. The initial learning rate is set to 10^−4^, which allows the network to converge stably, and is strategically reduced by a factor of 0.1 at the 8th and 11th epochs to fine-tune the optimization process and improve convergence in later stages.

To maintain consistency and fairness in performance evaluation, all additional experimental settings are meticulously aligned with those used for the ImVoxelNet baseline. This includes dataset preprocessing, data augmentation strategies, and training hyperparameters, which are kept identical to eliminate confounding variables. By standardizing these aspects, we ensure that any observed performance improvements are directly attributable to the innovations introduced in our framework rather than variations in the experimental setup.

This rigorous and systematic approach minimizes variability and establishes a robust foundation for comparative evaluation. It underscores the reliability of the results by isolating the impact of the proposed methods, thereby providing a clear and unbiased assessment of their effectiveness. Furthermore, the consistency in experimental design enhances the reproducibility of our findings, facilitating further validation and extension of the proposed framework by the research community.

#### Dataset.

We evaluate the proposed method on the ScanNet dataset [25], a comprehensive benchmark widely used for 3D semantic understanding in indoor environments. ScanNet offers dense 3D reconstructions of real-world indoor scenes derived from RGB-D video sequences, making it an ideal dataset for assessing the performance of 3D detection methods in complex, cluttered environments with diverse object arrangements and occlusions.

The dataset comprises 1,513 scans spanning over 700 unique indoor scenes, partitioned into a training set containing 1,201 scans and a validation set with 312 scans. In total, ScanNet provides more than 2.5 million RGB images, each accompanied by depth maps, intrinsic camera parameters, and camera pose information. Furthermore, the dataset includes reconstructed 3D point clouds with per-point semantic annotations, enabling robust evaluation of methods targeting 3D object detection, instance segmentation, and semantic understanding. This richness in multimodal data and scene diversity makes ScanNet a valuable resource for benchmarking 3D detection frameworks.

To ensure comparability with prior works, we adhere to the standard evaluation protocol introduced in VoteNet [26]. In this protocol, we estimate 3D bounding boxes from the semantic point clouds provided by the dataset. These bounding boxes are axis-aligned, which means that object rotation angles are not explicitly predicted. This axis-alignment simplifies the evaluation and aligns with common practices in indoor 3D detection, where the primary focus is on accurately determining the spatial extent and positions of objects rather than their orientations.

For a fair comparison with baseline methods, we retrain the ImVoxelNet framework [17] using the same training configuration as our proposed method. Specifically, both frameworks process batches of 15 images per iteration, ensuring consistency in input data handling and computational resource utilization. During testing, we evaluate the models on 50 views of the validation set to assess their performance comprehensively. This consistent evaluation methodology ensures a direct and unbiased comparison between the proposed approach and the baseline, highlighting the advantages introduced by our framework.

By utilizing ScanNet’s dense annotations and following established evaluation protocols, we rigorously demonstrate the effectiveness of our method in capturing spatial and semantic cues for 3D detection in complex real-world indoor scenes. This meticulous approach further solidifies the validity of our results and reinforces the practical relevance of the proposed method in real-world applications.

#### Metrics.

The comparative methods are evaluated using the Mean Average Precision (mAP) metric[27], followed [17,28,29], a widely adopted standard for quantifying the performance of object detection models. The mAP metric evaluates a model’s ability to accurately detect and localize objects by computing the average precision (AP) over a range of Intersection over Union (IoU) thresholds. In the context of 3D object detection, the IoU thresholds typically span from 0.25 to 0.50, reflecting the challenges associated with detecting objects in three-dimensional spaces, where precision in localization plays a critical role.

The use of multiple IoU thresholds ensures that the evaluation captures the model’s performance under varying levels of overlap between the predicted bounding boxes and the ground truth. Lower thresholds, such as 0.25, test the model’s ability to approximately localize objects, which can be important in scenarios where precise boundaries are less critical. Higher thresholds, such as 0.50, impose stricter requirements, assessing the model’s capacity for accurate spatial alignment and its robustness in challenging cases with cluttered or occluded objects.

By averaging the precision across these thresholds, the mAP metric provides a balanced and comprehensive assessment of a model’s detection capabilities. This approach ensures that the evaluation does not disproportionately favor models that excel only at coarse localization or, conversely, those that perform well solely under strict conditions. Instead, it reflects a model’s overall proficiency in detecting objects with diverse spatial alignments and degrees of overlap.

Furthermore, the mAP metric is particularly effective for 3D object detection tasks because it accommodates the inherent complexities of 3D data, such as variations in scale, occlusion, and perspective. It enables a reliable comparison of different detection methods by standardizing the evaluation process, thereby facilitating a clear understanding of each method’s strengths and weaknesses. By leveraging this robust metric, we ensure the fairness and validity of the comparative analysis, providing meaningful insights into the performance of the proposed framework relative to baseline approaches.

### Comparison and analyse

In Table 1, we present a detailed comparison of our proposed method against several state-of-the-art approaches. The table provides a breakdown of the Average Precision (AP) for 18 categories within the ScanNetV2 dataset, along with the mean Average Precision (mAP) at an IoU threshold of 0.25. This comprehensive evaluation highlights the strengths and weaknesses of our approach compared to competing methods.

**Table 1:** Quantitative result with multi-view RGB inputs is evaluated on the val-set of ScanNet-V2.

group	Methods	cab	bed	chair	sofa	tabl	door	wind	bkshf	pic	cntr	desk	curt	fridg	showr	toil	sink	bath	garb	mAP@.25
1	Seg-Cluster [31]	11.8	13.5	18.9	14.6	13.8	11.1	11.5	11.7	0.0	13.7	12.2	12.4	11.2	18.0	19.5	18.9	16.4	12.2	13.4
1	Mask R-CNN [32]	15.7	15.4	16.4	16.2	14.9	12.5	11.6	11.8	19.5	13.7	14.4	14.7	21.6	18.5	25.0	24.5	24.5	16.9	17.1
1	SGPN [31]	20.7	31.5	31.6	40.6	31.9	16.6	15.3	13.6	0.0	17.4	14.1	22.2	0.0	0.0	72.9	52.4	0.0	18.6	22.2
1	3D-SIS [33]	12.8	63.1	66.0	46.3	26.9	8.0	2.8	2.3	0.0	6.9	33.3	2.5	10.4	12.2	74.5	22.9	58.7	7.1	25.4
1	3D-SIS (w/ RGB) [33]	19.8	69.7	66.2	71.8	36.1	30.6	10.9	27.3	0.0	10.0	46.9	14.1	53.8	36.0	87.6	43.0	84.3	16.2	40.2
1	VoteNet [34]	36.3	87.9	88.7	89.6	58.8	47.3	38.1	44.6	7.8	56.1	71.7	47.2	45.4	57.1	94.9	54.7	92.1	37.2	58.7
1	FCAF3D [35]	57.2	87.0	95.0	92.3	70.3	61.1	60.2	64.5	29.9	64.3	71.5	60.1	52.4	83.9	99.9	84.7	86.6	65.4	71.5
1	CAGroup3D [36]	60.4	93.0	95.3	92.3	69.9	67.9	63.6	67.3	40.7	77.0	83.9	69.4	65.7	73.0	100.0	79.7	87.0	66.1	75.12
2	Nerf-Det [28]	32.7	84.7	74.6	62.7	52.7	35.1	17.7	48.4	0.0	49.8	64.6	18.5	60.3	48.3	90.7	51.0	76.8	30.4	50.1
2	NeRF-Det++ [29]	38.9	83.9	73.9	77.6	57.2	33.3	23.5	47.9	1.5	56.9	77.7	21.1	61.5	46.8	92.8	49.2	80.2	34.5	53.2
2	Nerf-DetS [30]	43.7	83.8	78.3	83.0	56.8	43.2	28.8	52.0	3.8	70.5	69.8	28.5	59.4	61.6	93.1	52.4	83.3	44.5	57.6
3	ImVoxelNet [17]	30.0	84.2	73.8	67.3	53.7	31.6	14.7	41.3	2.0	24.6	62.6	18.4	46.2	30.8	89.8	55.4	72.1	29.7	46.0
3	ours	32.7	82.9	74.4	70.2	56.1	33.6	16.0	39.3	2.0	38.8	69.5	14.7	58.5	24.5	92.6	54.9	72.8	33.3	48.2

The first section of the table presents methods based on point clouds, while the second section showcases methods based on RGB images. However, the methods in the second section incorporate depth information as supervision during the training phase. The remaining methods utilize pure RGB for both training and inference.

In Table 1, we present a detailed comparison of our proposed method against several state-of-the-art approaches. The table provides a breakdown of the Average Precision (AP) for 18 categories within the ScanNetV2 dataset, along with the mean Average Precision (mAP) at an IoU threshold of 0.25. This comprehensive evaluation highlights the strengths and weaknesses of our approach compared to competing methods.

The results in the first section of Table 1 correspond to methods that rely on 3D point clouds as input. These methods directly process 3D geometric information and output annotations on the reconstructed 3D point cloud. In contrast, the third section includes methods that utilize RGB images as input, which represents a fundamentally different problem setup. This distinction allows us to provide a more nuanced comparison, as our method exclusively relies on RGB images, requiring the network to infer 3D spatial structures indirectly from 2D visual data.

The results indicate that our algorithm achieves over 10% improvement in mAP compared to earlier point cloud-based methods (e.g. 3D-SIS[33]), demonstrating the robustness of our framework in extracting meaningful spatial information even without direct access to 3D point cloud data. However, our approach still shows certain limitations when compared to more advanced point cloud-based algorithms, which leverage explicit geometric and depth information to achieve superior performance. This disparity underscores the inherent challenges associated with relying solely on RGB images, as the absence of depth data forces the network to learn 3D spatial relationships implicitly. Tasks such as epipolar matching and spatial reasoning, which are naturally encoded in point cloud data, must be approximated through feature extraction in RGB-based methods. In addition, considering that point cloud data is difficult to obtain and that methods using depth cameras are inherently expensive in terms of equipment, utilizing multi-view reconstruction algorithms to generate point clouds would introduce additional computational burdens. Therefore, we believe that the trade-off between performance and computational efficiency should be evaluated based on the task itself. For typical household and civilian scenarios, multi-view methods are sufficient to meet the requirements.

Similarly, compared to methods in the second section of Table 1 that use depth information as supervision during the training phase, the algorithm proposed in this paper lags in performance due to its inability to explicitly learn and perceive scene depth. However, it also reduces the computational power requirements. We believe that this trade-off is acceptable.

Compared to the ImVoxelNet baseline, our approach demonstrates significant performance gains, particularly due to its enhanced ability to capture and leverage geometric perceptual features. This improvement validates the effectiveness of the design choices. By incorporating a more robust feature representation, our method better models the underlying spatial structures, resulting in more accurate object detection and localization.

**Fig 3:**
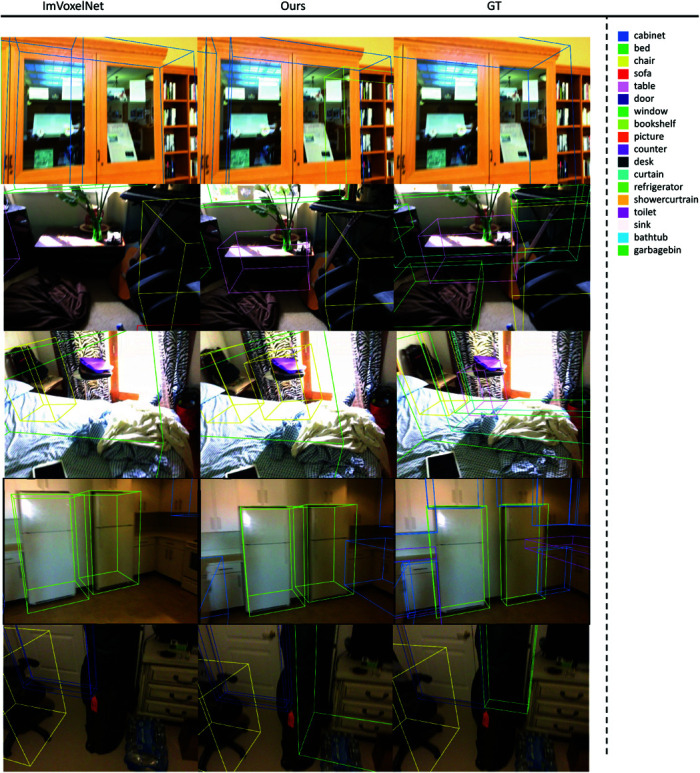
Visual comparison: We conduct a visual comparison between our and the current state-of-the-art method, ImVoxelNet [17], using the ScanNet [25] dataset. To illustrate the differences, we visualize randomly selected images from multiple scenes, providing a qualitative assessment of the detection performance and highlighting the advantages of our approach.

To further illustrate the effectiveness of our method, we include visual comparisons in Fig 3, showcasing detection results across different categories. For clarity, objects from various categories are visualized using distinct colors. We randomly select scenes from the dataset and provide example visualizations for each. These visualizations highlight the superior performance of our method in detecting and localizing objects, particularly those missed by baseline models. Notably, our approach excels in identifying objects with challenging spatial arrangements and occlusions, providing more accurate positions and bounding boxes compared to baseline methods.

In the figure, our methods gains: 1. Improved Detection Accuracy: Our method demonstrates the ability to detect missing objects that are overlooked by baseline models, resulting in more comprehensive scene understanding. 2. Superior Localization: The positional accuracy of detected objects is significantly enhanced, as evidenced by the visual results and the detailed breakdown of AP across categories.

The limitations of using pure RGB data are apparent when compared to advanced point cloud-based methods. However, the performance gap is partially bridged through the enhanced perception module introduced in our framework. The results in Table 1 and Fig 3 provide strong evidence of the effectiveness of our approach. While the lack of explicit depth information presents inherent challenges, the improvements over baseline methods confirm the strength of our proposed framework. These findings also validate the hypothesis introduced in section of Back-projection, emphasizing the importance of enhanced feature perception in achieving accurate 3D object detection from RGB data.

### Ablation study

To validate the effectiveness of our ImVoxelGNet network structure and the correctness of the integration of its various modules, we perform a series of ablation experiments. These experiments are conducted on the ScanNetV2 [25] dataset.

We investigated the impact of the proposed modules on the model’s performance by conducting a series of experiments. First, we established a baseline model as the foundation for comparison, as shown in Group. baseline of Table 2. Our baseline is ImVoxelNet [17].

**Table 2:** Ablation results.

Group	Modules	mAP@0.25↑	mAP@0.5↑
baseline	ImVoxelNet[17]	46.0	23.8
a1	4-F	46.4	22.3
a2	8-F	45.9	23.1
a3	8-L	47.1	22.5
b	GIP	47.1	22.9
c	GIP+8L	48.1	24.6

We compared the detection results using different numbers of input images. The first section presents the results of the baseline method, while the lower two sections show the results of our proposed method. 4-F and 8-F denote the use of fixed convolutional kernels based on the four-neighborhood and eight-neighborhood, respectively. In contrast, 8-L represents the use of a learnable convolutional kernel based on the eight-neighborhood.

In group a, we investigate the influence of different feature expansion methdods.

- Experiment a1. We utilize a fixed convolutional 4-neighbor kernel where the top, bottom, left, and right neighbors each had a weight of 1/8, and the center pixel had a weight of 1/2.
- Experiment a2. We use a fixed convolutional 8-neighbor kernel where the center pixel had a weight of 1/2, and the eight surrounding pixels each had a weight of 1/16.
- Experiment a3. A learnable 8-neighbor convolutional kernel is employed, allowing the model to adaptively determine the optimal weights.

The results reveal a surprising observation: in Experiment a2, the performance declined compared to the baseline. This suggests that incorporating more pixel features indiscriminately does not necessarily lead to better results, as the distribution of relevant features may not align with such a strategy. Conversely, Experiment a3 demonstrated significant performance improvements, highlighting that a well-designed or learnable convolutional kernel can effectively enhance feature aggregation and boost model performance. We suggest that there are two main reasons why the 8-neighbor fixed-weight convolution kernel is not as effective as the learnable convolution kernel. The first one is that learnable convolutional kernels allow the model to adapt to the specific data and task. The second is that Learnable filters can represent a wide range of complex patterns in the input data, while manually defined filters are often limited in terms of the types of patterns they can capture.

In Experiment of group b, we observed that incorporating implicit geometry awareness also resulted in a noticeable performance improvement. This enhancement can be attributed to the better geometric perception provided by this approach. Finally, by combining both 8-neighbor learnable contributions—adaptive convolutional kernels and implicit geometry awareness—we achieved the best performance improvement, demonstrating the full potential of our proposed method.

Overall, these ablation studies effectively validate the effectiveness of our approach, highlighting how each component contributes to the model’s performance and how their integration leads to optimal results.

As shown in Table 3, in another set of ablation experiments, we use Nerf-Det as the baseline and incorporate the proposed eight-neighborhood feature expansion module, comparing it with the baseline model. The results reveal that, since Nerf-Det itself uses depth information as supervision, the baseline performance is better than our ImVoxelGNet. However, after adding the feature expansion module in the projection phase, the performance of Nerf-Det still improves, which sufficiently demonstrates the effectiveness of the eight-neighborhood feature expansion module and validates the innovation of this paper. Similarly, since depth information is already used as explicit supervision, no comparison with the GIP module is conducted.

**Table 3:** Ablation results on Nerf-Det [28].

Group	modules	mAP@0.25↑	mAP@0.5 ↑
baseline	Nerf-Det [28]	50.1	25.4
a	Nerf-Det+8-L	51.2	25.9

We also conducted an ablation study on the NeRF-Det baseline. We incorporated the proposed 8L feature expansion module into the projection phase of NeRF-Det and compared it with the original NeRF-Det. The results show that the introduction of this module leads to an improvement in performance.

Furthermore, we conduct a set of ablation experiments to compare the robustness to noise. We added noise with a mean of 0 and different standard deviations to both the baseline and our ImVoxelGNet to test the network’s response to various levels of noise. The detailed results can be found in Table 4. As observed, both methods experience some performance degradation with the increase in noise. However, due to the presence of the pre-processing eight-neighborhood convolutional kernel, the proposed method demonstrates better resistance to noise and maintains its lead, thereby validating the robustness of the algorithm against noise.

**Table 4:** Ablation results on noise.

Group	std	mAP@0.25↑	mAP@0.5 ↑
baseline [17]	0	46.0	23.8
baseline [17]	0.1	43.9	21.6
baseline [17]	0.2	39.4	18.81
ours	0	48.1	24.6
ours	0.1	46.2	22.9
ours	0.2	41.9	19.3

We introduce Gaussian noise with different standard deviations and compare the inference results between the baseline and ImVoxelGNet. The proposed algorithm demonstrates better robustness against noise.

### Complexity analysis

We additionally tested the complexity of the network and the inference time of the network. Furthermore, we compared these results with a detection algorithm that only uses RGB.

We have included a comparison experiment regarding computational efficiency. Unlike the training phase, which utilized four GTX 1080 Ti GPUs, we used a mobile GTX 1070 for the time efficiency analysis to validate the potential of the proposed algorithm in edge computing, VR, and other fields. It is important to note that the computing power of the GTX 1070 is only about 40% of the floating-point performance of the current mainstream consumer platform, the RTX 4060, which further demonstrates the effectiveness of the algorithm proposed in this paper.

As shown in Table 5, the inference time for each image is 0.19 seconds, which is only 0.01 seconds longer than the baseline, yet the performance improvement is highly significant. Furthermore, in terms of parameters, since we primarily added a few 3D and 2D convolutional kernels, the proposed method only increases the parameter count by 51K, resulting in a 2.1 mAP improvement. We believe this represents a very worthwhile trade-off between parameter count and performance.

**Table 5:** Complexity comparison results.

Method	Paramater	Time Cost(s)	mAP@0.25↑
ImVoxelNet	104.5M	0.18s	46.0
ours	104.5M(+51K)	0.19s	48.1

We conduct a comparison in terms of parameter and time efficiency, performing inference experiments on a GTX 1070 mobile graphic card. The inference time for a single image is approximately 0.19 seconds. The increase in the number of network parameters is minimal, as it is primarily attributed to the parameters associated with the GIP module and the 8L module, with about 51K increase in parameter count being very small.

## Conclusion

In this paper, we address the task of 3D object detection based on geometric images. Specifically, we focus on improving the back-projection process, a common approach in current algorithms, which often suffers from limitations in accurately and comprehensively mapping pixels to voxels. Our proposed method enhances the perception of potential 3D geometric structures by introducing a more robust and precise mapping strategy. Additionally, we incorporate an implicit voxel occupancy classification module that leverages spatial geometric information. The classification results are subsequently updated into the voxel space, further enhancing the network’s understanding of 3D structures. Through these innovations, our method achieves significant improvements in overall network performance and produces superior results.

### Limitations and future work

From Table 1, we can observe that the algorithm proposed in this paper demonstrates some improvement in detecting small objects. However, for larger object categories, such as beds and bookshelves, the detection results show a slight decline. We attribute this to the fact that the proposed algorithm takes full advantage of pixel and implicit geometric information that was not fully utilized in previous baselines, leading to improvements. These contributions are primarily in the use of local information, while the broader range of information has not been utilized as effectively. This is reflected in our approach, which makes additional use of pixels in the eight-neighborhood surrounding the projected pixels in Fig 2, rather than combining and using object-level or semantic-level clues as priors.

Therefore, our goal for future tasks is to employ more flexible variable convolutions and explore ways to improve the projection algorithm, in order to fully leverage every foreground object pixel and its geometric features. This approach aims to further enhance the results of 3D detection tasks.

## References

[1] Naseer M, Khan S, Porikli F. Indoor scene understanding in 2.5/3D for autonomous agents: a survey. IEEE Access. 2019;7:1859–87. doi: 10.1109/access.2018.2886133

[2] Zhang Y, Song S, Yumer E, Savva M, Lee J, Jin H. Physically-based rendering for indoor scene understanding using convolutional neural networks. In: Proceedings of the IEEE conference on computer vision and pattern recognition; 2017. pp. 5287–95.

[3] Baruch G, Chen Z, Dehghan A, Dimry T, Feigin Y, Fu P. Arkitscenes: A diverse real-world dataset for 3D indoor scene understanding using mobile rgb-d data. arXiv. 2021. doi: arXiv:2111.08897

[4] Nie Y, Han X, Guo S, Zheng Y, Chang J, Zhang J. Total 3D understanding: Joint layout, object pose and mesh reconstruction for indoor scenes from a single image. In: Proceedings of the IEEE/CVF conference on computer vision and pattern recognition. 2020. pp. 55–64.

[5] Mao J, Niu M, Bai H, Liang X, Xu H, Xu C. Pyramid r-cnn: Towards better performance and adaptability for 3D object detection. In: Proceedings of the IEEE/CVF international conference on computer vision. 2021. pp. 2723–32.

[6] Yin T, Zhou X, Krahenbuhl P. Center-based 3D object detection and tracking. In: Proceedings of the IEEE/CVF conference on computer vision and pattern recognition. 2021. pp. 11784–93.

[7] Huang S, Qi S, Xiao Y, Zhu Y, Wu YN, Zhu SC. Cooperative holistic scene understanding: unifying 3D object, layout, and camera pose estimation. Adv Neural Inform Process Syst. 2018;31.

[8] Su H, Maji S, Kalogerakis E, Learned-Miller E. Multi-view convolutional neural networks for 3D shape recognition. In: Proceedings of the IEEE international conference on computer vision. 2015. pp. 945–53.

[9] Zhou Q, Cao J, Leng H, Yin Y, Kun Y, Zimmermann R. SOGDet: Semantic-occupancy guided multi-view 3D object detection. AAAI. 2024;38(7):7668–76. doi: 10.1609/aaai.v38i7.28600

[10] Yang C, Zheng J, Dai X, Tang R, Ma Y, Yuan X. Learning to reconstruct 3D non-cuboid room layout from a single rgb image. In: Proceedings of the IEEE/CVF winter conference on applications of computer vision. 2022. pp. 2534–43.

[11] Lee CY, Badrinarayanan V, Malisiewicz T, Rabinovich A. Roomnet: End-to-end room layout estimation. In: Proceedings of the IEEE international conference on computer vision. 2017. pp. 4865–74.

[12] Zhang C, Cui Z, Zhang Y, Zeng B, Pollefeys M, Liu S. Holistic 3D scene understanding from a single image with implicit representation. In: Proceedings of the IEEE/CVF conference on computer vision and pattern recognition. 2021. pp. 8833–42.

[13] Murez Z, van As T, Bartolozzi J, Sinha A, Badrinarayanan V, Rabinovich A. Atlas: End-to-end 3D scene reconstruction from posed images. In: ECCV. 2020. Available from: https://arxiv.org/abs/2003.10432

[14] Zhu Z, Peng S, Larsson V, Xu W, Bao H, Cui Z. Nice-slam: Neural implicit scalable encoding for slam. In: Proceedings of the IEEE/CVF conference on computer vision and pattern recognition. 2022. pp. 12786–96.

[15] Facil JM, Concha A, Montesano L, Civera J. Single-view and multi-view depth fusion. IEEE Robot Autom Lett. 2017;2(4):1994–2001. doi: 10.1109/lra.2017.2715400

[16] Yu A, Ye V, Tancik M, Kanazawa A. pixelnerf: Neural radiance fields from one or few images. In: Proceedings of the IEEE/CVF conference on computer vision and pattern recognition. 2021. pp. 4578–87.

[17] Rukhovich D, Vorontsova A, Konushin A. Imvoxelnet: Image to voxels projection for monocular and multi-view general-purpose 3D object detection. In: Proceedings of the IEEE/CVF winter conference on applications of computer vision. 2022. pp. 2397–406.

[18] Jiang X, Li S, Liu Y, Wang S, Jia F, Wang T. Far3D: Expanding the horizon for surround-view 3D object detection. arXiv preprint. 2023. doi: arXiv:2308.09616

[19] Mao J, Shi S, Wang X, Li H. 3D object detection for autonomous driving: a comprehensive survey. Int J Comput Vis. 2023;131(8):1909–63. doi: 10.1007/s11263-023-01790-1

[20] Yu Z, Liu Q, Wang W, Zhang L, Zhao X. PolarBEVDet: Exploring polar representation for multi-view 3D object detection in bird’s-eye-view. arXiv preprint. 2024. doi: arXiv:240816200

[21] Lang AH, Vora S, Caesar H, Zhou L, Yang J, Beijbom O. Pointpillars: Fast encoders for object detection from point clouds. In: Proceedings of the IEEE/CVF conference on computer vision and pattern recognition. 2019. pp. 12697–705.

[22] Yan Y, Mao Y, Li B. SECOND: Sparsely embedded convolutional detection. Sensors (Basel). 2018;18(10):3337. doi: 10.3390/s18103337 30301196 PMC6210968

[23] Zhang Z, Sun B, Yang H, Huang Q. H3dnet: 3D object detection using hybrid geometric primitives. In: European conference on computer vision. 2020. p. 311–29.

[24] Chen K, Wang J, Pang J, Cao Y, Xiong Y, Li X. MMDetection: Open mmlab detection toolbox and benchmark. arXiv preprint. 2019. doi: 10.48550/arXiv.1906.07155

[25] Dai A, Chang AX, Savva M, Halber M, Funkhouser T, Nießner M. Scannet: Richly-annotated 3D reconstructions of indoor scenes. In: Proceedings of the IEEE conference on computer vision and pattern recognition. 2017. pp. 5828–39.

[26] Qi C, Litany O, He K, Guibas LJ. Deep hough voting for 3D object detection in point clouds. In: Proceedings of the IEEE/CVF international conference on computer vision. 2019. pp. 9277–86.

[27] Everingham M, Van Gool L, Williams CKI, Winn J, Zisserman A. The Pascal visual object classes (VOC) challenge. Int J Comput Vis. 2009;88(2):303–38. doi: 10.1007/s11263-009-0275-4

[28] Xu C, Wu B, Hou J, Tsai S, Li R, Wang J, et al. Nerf-det: Learning geometry-aware volumetric representation for multi-view 3D object detection. In: Proceedings of the IEEE/CVF international conference on computer vision. 2023. p. 23320–30.

[29] Huang C, Hou Y, Ye W, Huang D, Huang X, Lin B. Nerf-det: Incorporating semantic cues and perspective-aware depth supervision for indoor multi-view 3D detection. arXiv preprint. 2024. doi: arXiv:240214464 10.1109/TIP.2025.3560240 40244844

[30] Huang C, Li X, Zhang S, Cao L, Ji R. NeRF-DetS: Enhancing multi-view 3D object detection with sampling-adaptive network of continuous NeRF-based representation. arXiv preprint. 2024. doi: 10.48550/arXiv.240413921

[31] Wang W, Yu R, Huang Q, Neumann U. Sgpn: Similarity group proposal network for 3D point cloud instance segmentation. In: Proceedings of the IEEE conference on computer vision and pattern recognition (CVPR). 2018.

[32] He K, Gkioxari G, Dollár P, Girshick R. Mask R-CNN. In: Proceedings of the IEEE international conference on computer vision (ICCV). 2017.

[33] Hou J, Dai A, Nießner M. 3D-SIS: 3D semantic instance segmentation of RGB-D scans. In: Proceedings of the IEEE conference on computer vision and pattern recognition (CVPR). 2019.

[34] Qi CR, Litany O, He K, Guibas LJ. Deep hough voting for 3D object detection in point clouds. In: Proceedings of the IEEE/CVF international conference on computer vision. 2019. pp. 9277–86.

[35] Rukhovich D, Vorontsova A, Konushin A. Fcaf3d: Fully convolutional anchor-free 3D object detection. In: European conference on computer vision. Springer; 2022. pp. 477–93.

[36] Wang H, Ding L, Dong S, Shi S, Li A, Li J, et al. CAGroup3D: Class-aware grouping for 3D object detection on point clouds. In: Oh AH, Agarwal A, Belgrave D, Cho K, editors. Advances in neural information processing systems. 2022.

